# Sublingual sugar for hypoglycaemia in children with severe malaria: A pilot clinical study

**DOI:** 10.1186/1475-2875-7-242

**Published:** 2008-11-23

**Authors:** Bertrand Graz, Moussa Dicko, Merlin L Willcox, Bernard Lambert, Jacques Falquet, Mathieu Forster, Sergio Giani, Chiaka Diakite, Eugène M Dembele, Drissa Diallo, Hubert Barennes

**Affiliations:** 1 Antenna Technologies, Genève, Switzerland; 2 University of Lausanne, Switzerland; 3 Département de Médecine Traditionnelle, Institut National de Recherche en Santé Publique, Bamako, Mali; 4 Hôpital de Sikasso, Sikasso Province, Mali; 5 Research Initiative on Traditional Antimalarial Methods, Oxford, UK; 6 Médecins du Monde, France; 7 Faculté de Médecine, Université de Laval, Québec, Canada; 8 Aidemet, Bamako, Mali; 9 Institut Francophone de Médecine tropicale, Vientiane, Laos; 10 Centre Muraz, Burkina Faso

## Abstract

**Background:**

Hypoglycaemia is a poor prognostic indicator in severe malaria. Intravenous infusions are rarely feasible in rural areas. The efficacy of sublingual sugar (SLS) was assessed in a pilot randomized controlled trial among hypoglycaemic children with severe malaria in Mali.

**Methods:**

Of 151 patients with presumed severe malaria, 23 children with blood glucose concentrations < 60 mg/dl (< 3.3 mmol/l) were assigned randomly to receive either intravenous 10% glucose (IVG; n = 9) or sublingual sugar (SLS; n = 14). In SLS, a teaspoon of sugar, moistened with a few drops of water, was gently placed under the tongue every 20 minutes. The child was put in the recovery position. Blood glucose concentration (BGC) was measured every 5–10 minutes for the first hour. All children were treated for malaria with intramuscular artemether. The primary outcome measure was treatment response, defined as reaching a BGC of >= 3.3 mmol/l (60 mg/dl) within 40 minutes after admission. Secondary outcome measures were early treatment response at 20 minutes, relapse (early and late), maximal BGC gain (CGmax), and treatment delay.

**Results:**

There was no significant difference between the groups in the primary outcome measure. Treatment response occurred in 71% and 67% for SLS and IVG, respectively. Among the responders, relapses occurred in 30% on SLS at 40 minutes and in 17% on IVG at 20 minutes. There was one fatality in each group. Treatment failures in the SLS group were related to children with clenched teeth or swallowing the sugar, whereas in the IVG group, they were due to unavoidable delays in beginning an infusion (median time 17.5 min (range 3–40).

Among SLS, the BGC increase was rapid among the nine patients who really kept the sugar sublingually. All but one increased their BGC by 10 minutes with a mean gain of 44 mg/dl (95%CI: 20.5–63.4).

**Conclusion:**

Sublingual sugar appears to be a child-friendly, well-tolerated and effective promising method of raising blood glucose in severely ill children. More frequent repeated doses are needed to prevent relapse. Children should be monitored for early swallowing which leads to delayed absorption, and in this case another dose of sugar should be given. Sublingual sugar could be proposed as an immediate "first aid" measure while awaiting intravenous glucose. In many cases it may avert the need for intravenous glucose.

## Background

Hypoglycaemia is common in tropical paediatrics [1] and is a poor prognostic indicator in severe malaria [2-4]. It often remains undetected in severely ill children. In Africa, many children with hypoglycaemia and severe malaria die before reaching a health care facility where intravenous treatment is available [5]. Intravenous infusions are rarely feasible in rural Africa because of the lack of simple equipment or trained staff and can be difficult to administer in young children[6]. Although intravenous infusions are not supposed to be given outside of secondary care settings, in practice they sometimes are (and sometimes abused or given unsafely [7]).

An alternative route of administration is via the sublingual mucosa and it is possible to give simple sugar rather than glucose, as shown previously among moderately hypoglycaemic children [8,9]. Sugar is degraded in the oral cavity by sucrase [10], then glucose is transported through the oral mucosa [11]; the transport system is more abundant under the tongue than in the buccal cavity [12]. This was a pilot study to assess the efficacy of sublingual sugar (SLS) in a randomized controlled trial among hypoglycaemic children with presumed severe malaria.

## Methods

### Setting

The study was conducted in the Paediatric Department of Sikasso Regional Hospital, in Southern Mali, during the peak malaria season (July – September 2006). All patients presenting with presumed severe malaria were screened for inclusion.

This 20-bed paediatric ward is staffed by one Malian consultant paediatrician (ED), two foreign doctors (Chinese and Cuban, neither of whom is a paediatrician), two or three interns, and six members of nursing staff (only one of whom is a fully qualified nurse). Out of hours there is one intern and one member of nursing staff on duty. There are 150–200 admissions per month during the peak malaria season, the majority with a presumed diagnosis of severe malaria. Case fatality rates prior to our study averaged 14–19% during these months [13].

### Participants

Children were eligible if they were aged 6 months to 15 years, met the WHO case definition of severe malaria [4], had seizures or were prostrated or in coma, had blood glucose concentration (BGC) < 60 mg/dl (< 3.3 mmol/l), and families gave oral and written informed consent. Children who required intravenous infusions for other reasons (treatment of shock, acidosis, transfusion) were not eligible.

Children were included in the analysis if sufficient blood glucose measurements were available to assign an outcome. Participants were randomised to receive sublingual sugar (SLS) or intravenous glucose (IVG), according to a pre-established randomisation list. Allocation was not concealed. Once the child was considered to comply with the inclusion criteria, s/he was allocated according to the randomised list under the supervision of the team.

### Interventions

The SLS group received a teaspoon of sugar (level teaspoon for those under 15 kilos, heaped teaspoon for those above that weight, which approximates to 2.5 g and 3.5 g of sugar, respectively) moistened with water placed under the tongue. Ordinary granulated sugar was used, bought from the local market. Treatment was repeated every 20 minutes and continued for two hours. After placing sugar under the tongue, children were placed in the recovery position to prevent inhalation. Furthermore, regular checks were made to see whether the sugar had been swallowed. SLS children who did not increase their BGC by 20 minutes or normalize their blood glucose by 40 minutes were switched to intravenous glucose.

The IVG group received 5 ml/kg 10% glucose intravenous infusion as soon as was practically possible. Conditions were artificially improved for provision of intravenous glucose. All the necessary equipment was provided free and was available for immediate use in the emergency room. All children were treated for malaria with intramuscular artemether. No patient was treated with quinine.

### Laboratory measurements

Before enrolment, 0.1 ml of blood was collected through finger prick to measure the baseline blood-glucose concentration (BGC). This was repeated every five minutes for 20 minutes, then every 10 minutes (Ascensia Esprit and Breeze glucometers, Bayer Diagnostics, precision of 1 mg/dl). Results were recorded in mg/dl (these can be converted to mmol/l, by dividing the result by 18: for example, 60 mg/dl = 3.3 mmol/l, 54 mg/dl = 3.0 mmol/l and 40 mg/dl = 2.2 mmol/L.

A thick film for malaria parasites was prepared and read afterwards. The definition of hypoglycemia was derived from standard paediatric textbooks: a blood glucose concentration < 60 mg/dl (< 3.3 mmol/L) was considered abnormally low and defined the hypoglycaemic group[14,15].

### Outcome measurements

One primary and four secondary outcome measurements were used.

The primary outcome was the treatment response rate, defined as reaching a glucose concentration over 60 mg/dl during the first 40 minutes. The following secondary outcomes were evaluated with the same method in both groups:

i) Early treatment response was defined as a significant blood glucose gain (> 10 mg/dl) at or before 20 minutes

ii) Relapse was defined as children who reached a normal glycaemia 3.3 mmol/L (60 mg/dl), but failed to maintain it during the study period defining early or late relapse (before 20 or after 20 minutes).

iii) The maximal BGC gain (CGmax) was defined as the difference between baseline BGC and the peak glucose concentration (on the first-line treatment) within the first 40 minutes.

iv) The treatment delay was the time from confirmation of hypoglycaemia to the beginning of treatment

### Statistical analysis

Analysis was performed on intention to treat. Data were entered in Epi-Info (version 6.04, CDC Atlanta). Since the interval between two BGC measurements was very short, some values were missing, they were treated as missing values. Hypoglycaemia over time was analysed according to the route of administration. Analysis was carried out by STATA, versions 8 (Stata Corporation, College Station, TX, USA). Fisher's exact tests were used for categorical variables, Student's t test for normally distributed continuous data, and Mann-Whitney test when appropriate. Probability values of < 0.05 were regarded as statistically significant. Results were expressed as mean and 95% confidence interval (95%CI) or median time and range as appropriate.

### Ethical considerations

The study was approved by the National Ethical Review Board of Mali. All patients' parents gave informed oral consent before being included in the study, which was then followed up with written consent. The study was performed in accordance with the Declaration of Helsinki [16].

## Results

Of 151 patients with presumed severe malaria between August and September 2006, 26 children (14 SLS, 12 IVG) met the inclusion criteria. Three IVG children were not tested for glycaemia during the follow-up due to a misunderstanding, (initially some staff thought that serial BGC measurements were only needed for patients on SLS), resulting in BGC data being available for 14 SLS and 9 IVG. These are the patients described in the analysis below.

Baseline characteristics are shown in Table 1. There was no clinically significant difference in the patients' characteristics between groups, except that there were more comatose children on IVG (p = 0.06).

**Table 1:** Characteristics of hypoglycaemic children (BGC < 60 mg/dl) on admission to Sikasso paediatric ward

	Sublingual Sugar (SLS) n = 14 (95%CI)	Intravenous Glucose (IVG) n = 9 (95%CI)
Sex (% F)	29	44
Mean age (months)	26.7 (19.8–33.7)	34.3 (5.8–62.8)
Mean weight (kg)	10.8 (9.4–12.7)	10.1 (6.9–13.1)
Duration of illness before presentation (days)	2.55 (1.4–4.1)	2.79 (1.8–3.1)
Coma^£^	2 (14%)	5 (56%)
Prostration (%)	12 (86%)	7 (78%)
Convulsions (%)	3 (21%)	1 (11%)
Respiratory distress (%)	7 (50%)	5 (56%)
Splenomegaly (%)	4 (29%)	4 (44%)
Malnutrition (%)*	1 (7%)	0
Mean temperature °C	38.9 (38.3–39.3)	38.3 (37.2–39.3)
Mean glycaemia (mg/dl)	46.5 (40.7–52.2)	45.0 (36.0–53.9)
Number of children with BGC < 40 mg/dl (< 2.2 mmol/l)	3 (21%)	2 (22%)
Number of children with BGC < 54 mg/dl (< 3.0 mml/L)	10 (71%)	6 (79%)
Thick film positive for *P. falciparum *(%)	11 (79%)	8 (89%)

^£ ^p = 0.06, The mean blood glucose was 50.0 and 47.6 mg/dl in the comatose children in groups SLS and IVG, respectively.

* underweight: weight for age below 2 standard deviation

Table 2 shows the outcomes by intention to treat analysis. There is no significant difference in the primary outcome measure between the groups. In the secondary outcome measures, there were more relapses in the SLS group, and treatment delay was much longer in the IVG group. There were no significant differences in the other secondary outcome measures. Only 2 SLS children were switched to an intravenous infusion within the 40 minutes after observed early swallowing and early failure at 20 minutes.

**Table 2:** Intention to treat analysis: Clinical and biological responses to IV glucose or sublingual sugar administration among children with hypoglycaemia (BGC < 60 mg/dl) in Mali

	Sublingual Sugar (SLS)	Intravenous Glucose (IVG)	*p*
	n = 14 (95%CI)	n = 9 (95%CI)	
Primary Outcome measure			
- Treatment Response (reaching 60 mg within 40 minutes)	10/14 (71%)	6/9 (67%)	0.81
			
Secondary outcome measures			
- Early treatment response^a^	9/14 (64.3%)	6/9 (67%)	0.91
- Relapse^b ^(as % of treatment responders)	3/10 (30%)	1/6 (17%)	0.55
-CGmax^d^(mg/dl)	43.4 (25.8–62.5)	46.2 (19.1–73.2)	0.60
-Treatment delay (mins)^e^	< 5	18.9 (6.4–31.6)	-
Case Fatality	1 (7%)	1 (11%)	NS

^a ^defined as a significant blood glucose gain (> 10 mg/dl) at or before 20 minutes

^b ^defined as children who reached a normal glycaemia 3.3 mmol/L (60 mg/dl), but failed to maintain it

^c ^defined as the difference between baseline BGC and the peak glucose concentration (on the first-line treatment) within the first 40 minutes

^d ^the time from confirmation of hypoglycaemia to the beginning of treatment. Treatment delay for SLS was always less than 5 minutes and was not recorded precisely so 5 minutes was taken as a conservative estimate; for IVG, treatment delay was the observed value.

About one-third of children had treatment failure in both groups. Treatment failures in the SLS group were related to children with clenched teeth or swallowing the sugar, whereas in the IVG group, they were due to unavoidable delays in beginning an infusion (median time 17.5 min (range 3–40).

The BGC increase was rapid in the SLS group with an overall mean gain of 36 mg/dl (95%CI:17.6–54.5) by 10 minutes. 8/14 (57%) increased their BGC significantly by 10 minutes (> 10 mg/dl). 9/14 (64%) had a BGC > 60 mg/dl by 20 minutes.

Relapse was more frequent in SLS group (30%). In the IVG group, there was one relapse at 20 minutes (BCG: 31 mg/dl) due to a blocked infusion, but the BGC had increased to 69 mg/dl by 40 minutes.

**Figure 1:**
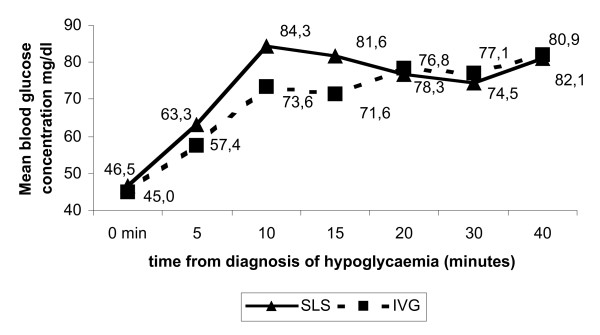
Mean blood glucose concentration in the sublingual (SLS) and dextrose infusion groups (IVG), by intention to treat.

During close monitoring, four children on SLS were detected to have swallowed the sugar rather than having kept it under their tongue. They had a slower BGC increase and two failed to reach 60 mg/dl at 40 minutes. They were given intravenous glucose (two at 20 minutes, two at 40 minutes). In contrast, eight of the nine patients who really kept the sugar sublingually increased their BGC during the follow-up. In this subgroup, all but one increased their BGC by 10 minutes, with a mean gain of 44 mg/dl (95%CI: 20.5–63.4). Figure 1 illustrates the speed with which BGC was corrected.

Glycaemia was slower to increase in IVG than in SLS, taking the start time as diagnosis of hypoglycaemia. Only four infusions were started in less than 10 minutes. The median time to begin an infusion was 17.5 minutes. Once the infusion was sited, BGC increased rapidly in all children in less than five minutes. One infusion became blocked after 15 minutes and the BGC dropped to 31 mg/dl.

SLS was well tolerated and no child developed any cough or symptoms suggesting inhalation. SLS was painless and easy to use in all children except for one with trismus.

## Discussion

This pilot study in a Malian district hospital reports the effect over a 40 minute period of two administrations of sublingual sugar in young children (< 5 years of age) with severe malaria and BGC below 60 mg/dl (3.3 mmol/l). This administration was compared with the standardised intravenous infusion of 10% glucose. Results were similar in both groups. Overall 71% of the SLS reached a BGC over 60 mg/dl within 40 minutes and 64% within 20 minutes. SLS resulted in a rapid correction of BGC; among nine children who really kept the sugar sublingually, all but one increased their BGC within 10 minutes of sublingual sugar administration with a mean gain of 44 mg/dl. These results suggest the efficacy of SLS for the rapid correction of hypoglycaemia, but also the need for more frequent and repeated administration as objectified by the 30% relapse rate at 40 minutes before the third administration of sugar. The number and interval of repeated sugar doses needed to obtain a steady BGC requires further study over a longer duration, and with more patients. The results of this study with children with BGC < 60 mg/dl give important information to allow a further study in children with more severe hypoglycaemia (e.g. < 40 mg/dl) and comatose children who stand to benefit more from such a treatment.

This study suggests that sublingual sugar is almost certainly better than no treatment because no significant gain in glycaemia was seen in patients in the IVG group before the intravenous infusion of glucose was started (and it would be unethical to include a no treatment arm in any study of hypoglycaemia in severe malaria). Therefore sublingual sugar can be recommended in situations where the gold standard treatment of intravenous glucose is not immediately available.

Hypoglycaemia is particularly common in children with falciparum malaria who are younger than three years of age [4,17,18]. This feature of severe malaria is associated with a three- to ten-fold increase in case fatality [3,19,23]. Correction of hypoglycaemia is an important therapeutic measure, although it is not clear whether this is sufficient to improve the prognosis.

When intravenous infusions are not feasible, because of lack of material or trained staff, which is the case in many rural health centres, no alternative has yet been developed. Oral absorption is delayed until the sugar reaches the duodenum [17] and may cause severe inhalation in prostrated or comatose children. The oral mucosa and the sublingual cavity offer a relatively unexplored alternative [8] though degradation of sucrose and a transport system of sucrose has been shown in the dorsal part of the tongue [11,12]. The previous study conducted in Burkina Faso in children with low glycaemia confirmed that SLS was more rapidly absorbed than oral sugar [8]. It also suggested the need for repeated administration and gave preliminary information on the interval doses for sugar administration. Further evaluation is needed to determine whether the interval can be decreased between administrations of sugar, which may enhance the efficacy of the SLS. Absorption appeared to be effective within the 10 first minutes in the present Malian study.

Compared to the previous study [6], the treatment failure and early failure rates were greater, but the CGmax was the same (46 mg/dl). This may be explained by the lower initial glycaemia of children in this study, their younger age and their more severe disease.

As in Burkina Faso SLS was well tolerated, painless and easy to use, no inhalation was reported. The feasibility was good with only one failure due to trismus. SLS appeared to be a child-friendly method.

Various definitions of hypoglycemic blood sugar levels for infants and children have been reported and remain controversial [20,21]. For severe malaria a cut-off of 2.2 mmol/L (40 mg/dl) is widely used but other thresholds have also been used: 41, 48, and 54 mg/dl [4,17,22,23]. The definition of standard paediatric reference texts was used in this study: the normal serum glucose concentration for children beyond the newborn period is greater than or equal to 60 mg/dl (3.3 mmol/L) [14,15]. In fact, the 3.0 mmol/L (54 mg/dl) cut-off could have been used with very similar results, since five children had a BGC of 55 mg/dl and two had a BGC of 57 mg/dl, with an equal distribution between both groups.

Interestingly, with sublingual sugar, the correction of hypoglycaemia tended to occur faster than with intravenous glucose because there are inevitable delays in setting up the infusion, as it is often difficult to find a vein in a small, unconscious and shocked child. Four early failures at 20 minutes were observed due to delays in the IVG group.

As in many West African hospitals, intra-osseous infusions are not available to avoid this delay. This should be an important improvement at the district level in the future if the necessary equipment, hygienic conditions and trained staff are available.

In this study results for IVG were artificially good, because all the equipment was made immediately available at the point of need, and free-of-charge. The normal situation in the hospital is that the patient's parents are given a prescription for the bag of intravenous glucose, the intravenous catheter, the administration set and any other necessary medications. They must then go and purchase this from the pharmacy, and bring it back to the ward before treatment can commence. This can introduce severe delays if the parent has to go and find or borrow the money, or if the pharmacy is out-of-stock of one or more items, which is not an infrequent occurrence. The advantage of sublingual sugar is that it is virtually free, immediately available, and does not require a prescription or a pharmacy. Furthermore, it is also available everywhere, including peripheral health centres and villages, where intravenous glucose is not available.

This study may have important implications for the clinical management of malaria in children with a potential risk of hypoglycaemia in the field. SLS could be used as a preventive intervention at home, but also at the health centre. In fact, use of SLS was rapidly adopted by the community in Sikasso region before and during referral of children. A community-based study could evaluate the impact of such a recommendation on the prevalence of hypoglycaemia and mortality of children admitted to hospital.

### Limitations

This study was performed in the field conditions of a busy West African paediatric ward, during the malaria season, which explains some of its limitations (the small sample size and the incomplete data for some patients).

In the SLS group, the number of comatose children was small and lower than in the IVG group (table 1). Most children had other features of severe malaria with respiratory distress, prostration and convulsions. Children in coma are the most likely to benefit from SLS administration and these preliminary results need to be confirmed in a larger scale study of comatose children.

The sublingual sugar administration has two main limitations. Firstly, at least 29% of the children swallowed the sugar rather than keeping it under the tongue (particularly children in a less severe state and hence able to swallow). The oral route has a slower absorption in the duodenum and is slower to normalize glycaemia [17]. Early swallowing may remain undetected by busy staff who have to be trained to detect this. When sugar is swallowed before 10 minutes, another SLS administration should be recommended. The non-responsive child on SLS may have swallowed the sugar undetected by the team. He received an infusion after 40 minutes so it is impossible to know whether he had delayed oral absorption. Secondly, the small volume of the sublingual cavity limits the SLS dose available and may restrict SLS to young children. Children were below five years of age and the SLS doses were satisfactory. However, this is the age group at greatest risk of severe malaria in Africa. The initial mean single SLS dose was 0.25 g/kg (95%CI: 0.20–1.28), which is below the recommended dose (0.5 g/kg). This implies the need to frequently repeat the SLS doses [7], in order to avoid relapses.

The limited duration time of the study only allowed evaluation of two administrations of sugar (i.e 5–6 g), but 3 or 4 administrations would be needed to obtain a steady BGC. Relapses at 40 minutes were observed after a rapid initial increase of BGC and BGC should be monitored until it remains stable [17] – or, when monitoring is impossible, SLS administration should be repeated every 10–20 minutes (e.g. during the transport to hospital). More studies with older children are necessary to see whether the SLS route is also adequate for older ages and how doses need to be adapted.

The aetiology of hypoglycaemia was presumed to be malaria and the duration of illness prior to the admission. However, the study did not enquire about other causes of hypoglycaemia and it is not known whether some traditional antimalarials may cause hypoglycaemia.

### Implications

Although the sample size is small, the results indicate sufficient safety and efficacy to justify the use of sublingual sugar at the community level and to justify a further larger trial. Intravenous treatment is often considered the gold standard. It may give a false sense of security when monitoring is not correct, which is often the case in remote settings. One infusion was blocked at 15 minutes and the child's BGC quickly dropped to a very low level.

In hospital, SLS can be recommended when infusions are not immediately available (or until they are put in place) or when a transfusion is urgently needed. In the case of parallel blood transfusions (patients with anaemia and hypoglycaemia), SLS carries a lower risk of fluid overload than IVG, and as such might be useful even in well resourced settings.

## Conclusion

This preliminary study suggests that sublingual sugar is a promising treatment for the prevention and correction of hypoglycaemia in children with severe malaria. Our results suggest that a more frequent repetition of doses is needed to prevent relapse, for example every 10 minutes instead of every 20 minutes. A larger study is needed to evaluate the more frequent dosing, and to confirm these results. This treatment is feasible in most children, even if comatose, as a "first-aid" measure by health care workers and even families in remote areas. This may impact on morbidity and mortality in children with hypoglycaemia, not only linked to severe malaria but also to malnutrition or intoxication, and has important implications for the clinical management of children in tropical countries.

## List of abbreviations

95%CI: 95% confidence interval; SLS: sublingual sugar; SLS: sublingual sugar group; BGC: blood glucose concentration; OS: oral sugar group; IVG: intravenous glucose group; CGmax: the maximal BGC gain; mg/dl: milligrammes per decilitre; mmol/l: Millimoles per liter; g/kg: Grammes per kilogramme.

## Authors' contributions

BG, HB, MLW, JF, SG, CD, DD and EMD developed the study design.  MD, BL, MLW, BG, EMD participated in the study conduct  HB, MLW, BG performed the analysis and interpretation of the data and    received critical comments from MF.  BG, HB, MLW wrote the manuscript, and received comments from the whole    research team prior manuscript submission. All authors read and approved the final version of the manuscript.
